# Special Issue “Nutritional Regulation on Gut Microbiota”: Editorial

**DOI:** 10.3390/microorganisms11020530

**Published:** 2023-02-19

**Authors:** Garry X. Shen

**Affiliations:** Departments of Internal Medicine, Food and Human Nutritional Science, University of Manitoba, 835-715 McDermot Ave, Winnipeg, MB R3E 3P4, Canada; garry.shen@umanitoba.ca; Tel.: +204-789-3816; Fax: +204-789-3987

Accumulated lines of evidence demonstrate that the gut microbiota plays a critical role in metabolism, inflammation and the pathophysiology of many chronic diseases [1]. Foods are the most important regulators for the growth of microorganisms in the gastrointestinal tract. The assessment of the composition of gut microbiota and their products may help determine the mechanism and impact of foods or nutrients on health. This Special Issue collected five research articles from worldwide investigators on the effects of different types of food products, nutrients or supplements with respect to the abundance of gut bacteria, relevant products and health-related biochemical variables in human subjects, cultured feces from human donors and animal models [2].

The studies examined the effects of three types of fruit products [3,4,5], a vegetable component extract [6] and a synthesized artificial sweetener [7] on gut microbiota. Among them, two studies were conducted in animal models [3,4], one study was a controlled clinical trial in healthy human subjects, and the other two were conducted in cultured feces from human donors [5,6,7].

Szmigiel et al. [3] found that fermented rapeseed meals increased the abundance of probiotic bacteria and inhibited pathogenic bacteria in feces from broiled chickens, which was associated with the improved histological structure of cecum epithelial cells. The findings may help chicken farmers to use fermented rapeseed meals as feeding components to reduce the requirement of antibiotics, which may benefit the health of humans who eat chicken products. Zhao et al. [4] demonstrated that the supplementation of 1–5% of Saskatoon berry powder (SBp) dose-dependently inhibited fasting plasma glucose and insulin; the homeostatic model assessment of insulin resistance, lipids and inflammatory markers in high fat with high sucrose (HFHS) diet induced insulin-resistant mice compared to that fed with HFHS diet alone. In addition, SBp supplementation increased the abundance of *Bacteroidetes* phylum bacteria and *Muribaculaceae* family bacteria. Recent studies suggested that the abundance of *Muribaculaceae* in HFHS diet-fed mice was associated with the concentrations of short-chain fatty acids (SCFA) in feces [8]. The findings may help define proper therapeutic regimens for using SBp to prevent diabetes and inflammation via the modification of gut microbiota in mice and potentially in humans.

Two studies assessed the generation of SCFA and the composition of bacteria in cultured feces from human donors treated with Baobab fruit pulp powder (BFPP) [5] or carrot-derived peptin extracts enriched with rhamnogaloacturonan (cRG-1) [6]. BFPP and cRG-1 increased the generation of acetate and propionate, and the abundance of *Bacteroidetes* in cultured feces [5,6]. The results suggest that BFPP and cRG-1 exhibit prebiotic potential in terms of SCFA generation. Méndez-Garcia et al. [7] examined the effects of the oral intake of sucralose, an artificial sweetener, in water for 10 weeks in 20 healthy subjects using the oral glucose tolerance test relative to insulin and fecal probiotic bacteria compared to a control group receiving a vehicle without sucralose addition. The results demonstrated that sucralose increased the levels of insulin and the areas of glucose under the curve, but it reduced the abundance of *Lactobacillus acidophilus* in feces compared to that in the subjects of the vehicle–control group [7]. The finding suggests that sucralose enhanced insulin resistance in humans, which may result from its inhibition on probiotic bacteria in the gut.

The research findings in this Special Issue suggested that many foods, nutrients and supplements regulate the composition of gut microbiota, which may alter gastrointestinal integrity and SCFA production, and regulate glucose/lipid metabolism, inflammatory mediators or insulin resistance in animals or humans. The gut microbiota may become a useful biological marker or target for nutritional interventions. The high-throughput data generated from microbiome studies have not been made the best use of in most cases. Recent studies suggest that the incorporation of machine learning and artificial intelligence techniques in the analyses of gut microbiota, metabolomics and clinical data may provide insight into the interactions between bacteria, host and nutrients and predict effective dietary interventions [9,10]. An investigation on the nutritional regulation of the gut microbiota may help design a healthy diet for improving health and for preventing chronic metabolic or inflammatory diseases.
